# Hierarchical honeycomb auxetic metamaterials

**DOI:** 10.1038/srep18306

**Published:** 2015-12-16

**Authors:** Davood Mousanezhad, Sahab Babaee, Hamid Ebrahimi, Ranajay Ghosh, Abdelmagid Salem Hamouda, Katia Bertoldi, Ashkan Vaziri

**Affiliations:** 1 Department of Mechanical and Industrial Engineering, Northeastern University, Boston, MA 02115, USA; 2 Harvard John A. Paulson School of Engineering and Applied Sciences, Harvard University, Cambridge, MA 02138, USA; 3 Mechanical and Industrial Engineering Department, Qatar University, Doha, Qatar

## Abstract

Most conventional materials expand in transverse directions when they are compressed uniaxially resulting in the familiar positive Poisson’s ratio. Here we develop a new class of two dimensional (2D) metamaterials with negative Poisson’s ratio that contract in transverse directions under uniaxial compressive loads leading to auxeticity. This is achieved through mechanical instabilities (i.e., buckling) introduced by structural hierarchy and retained over a wide range of applied compression. This unusual behavior is demonstrated experimentally and analyzed computationally. The work provides new insights into the role of structural organization and hierarchy in designing 2D auxetic metamaterials, and new opportunities for developing energy absorbing materials, tunable membrane filters, and acoustic dampeners.

In recent years, synthetic metamaterials with negative Poisson’s ratio (defined as the negative of the ratio between transverse and longitudinal strains in uniaxial elastic loading) have been proposed^1^,^2^,^3^,^4^,^5^,^6^. In contrast to conventional materials, these so-called “auxetic” metamaterials contract in the transverse directions when compressed uniaxially^7^. This behavior is usually linked to specific microstructural deformation mechanisms also observed in traditional auxetic structures such as re-entrant, chiral, and rotating-units structures^8^,^9^,^10^,^11^,^12^,^13^,^14^,^15^,^16^. On the other hand, elastic instability (i.e., buckling) can also be utilized to induce auxetic behavior over a wide range of applied strains in the structures, which otherwise show positive Poisson’s ratio at small deformations^17^,^18^,^19^. Particularly, in this context, the role of hierarchy has been recently explored by Mousanezhad *et al.*^17^ who demonstrated auxetic behavior in a hierarchical “spiderweb” honeycomb at large deformations through a combination of numerical simulations and experiments.

Here, we exploit elastic instabilities along with structural hierarchy to design a new class of 2D auxetic metamaterials capable of exhibiting negative Poisson’s ratio over a wide range of applied compressive strains. Our study shows that the origin of this behavior is linked to the added hexagonal features within the hierarchical structure which make the instabilities to occur at smaller compressive strains compared to the original non-hierarchical structure leading to auxeticity. In fact, these particular buckling modes have been previously observed in regular hexagonal honeycombs but they did not lead to auxeticity^20^.

The hierarchical structure studied in this article which was first introduced by Ajdari *et al.*^21^, exhibited higher stiffness and more phononic bandgaps compared to its regular non-hierarchical counterpart^21^,^22^,^23^. The structure exhibits a positive Poisson’s ratio, ranging from ~0.37 to 1, at small deformations for first order of hierarchy^21^. The first level of hierarchy which was achieved by replacing the vertices of a regular hexagonal lattice with smaller hexagons and reducing the wall thickness to keep the overall density fixed, could be repeated to reach higher levels of hierarchy. Figure 1 shows the evolution of a regular hexagonal honeycomb and its corresponding cell as the order of hierarchy is increased. The geometrical organization of this structure at each order of hierarchy (*γ*_*i*_) is defined by the ratio of the newly added hexagonal edge length (*b* for first order and *c* for second order of hierarchy, see Fig. 1) to the original hexagon’s edge length (*a*) (i.e., 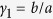, and 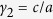)^21^ (See Supplementary Information for more details). The density of the structure (i.e., area fraction) normalized by the parent material density can be given as^21^


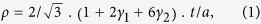


where *t* is the wall thickness which is assumed to be uniform throughout the structure.

## Results

**Figure 2:**
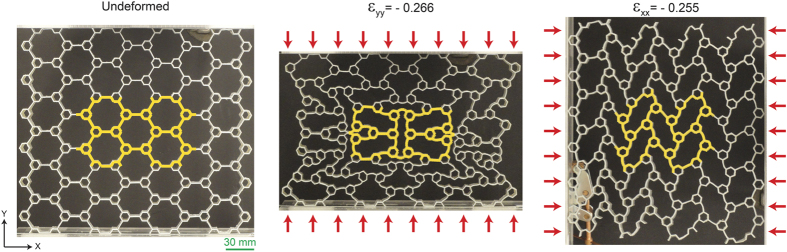
Hierarchical honeycomb auxetic metamaterials. (left) Undeformed configuration of the fabricated first order hierarchical structure with 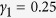. The representative volume element (RVE) is highlighted as yellow. (middle and right) Deformed configurations of the specimen and the RVE under compression along the *y* (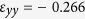, X-shape deformation mode) and *x* (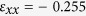, N-shape deformation mode) directions, respectively.

We subjected a specimen with first order of hierarchy (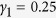) under uniaxial compression along the *y* and *x* directions (see Methods and Supplementary Information for more details), Fig. 2. The response of the specimen was monitored by taking photographs at different levels of compression with local strains 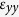 and 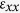 in either direction measured within the inner-most unit of the specimen (i.e., Representative Volume Element (RVE), highlighted in yellow in Fig. 2) to avoid boundary effects^18^,^24^,^25^. Note that the classical definition of an RVE relies on a limit of relatively infinite size of the sample thereby making boundaries irrelevant. However, finite sample size is inevitable in experiments and in the current work we seek to minimize the boundary effects on the inner-most unit of the specimen by choosing sufficient numbers of unit cells in the test sample thereby making it equivalent to an RVE in an infinite periodic media. Thus, in this work, we compare the experimental response of only the inner-most unit cell, which has minimal boundary effects with our numerical simulations, which rely on the assumption of an infinite periodic sample (the details of numerical simulations will be explained shortly). Figure 2 shows the undeformed and deformed configurations of the specimen and its RVE. As the deformation proceeds in either direction, the lateral sides of the specimen bulge inward, showing a perceptible 2D auxetic behavior which has not been observed in the non-hierarchical counterpart. Interestingly, two different types of deformation mode (i.e., buckling mode) are identified depending on the direction of the applied compression: X-shape and N-shape modes, respectively for the *y* and *x* direction loads. In the X-shape mode, the RVE’s deformation is mostly governed by elastic buckling of the horizontal cell walls and rotation of the corresponding smaller hexagons in the central hexagonal cell, while the RVE’s other horizontal cell walls and corresponding smaller hexagons remain almost intact. This is analogous to the buckling mode of a regular hexagonal honeycomb in biaxial compression^20^,^26^. The N-shape deformation mode on the other hand, is characterized by a zigzag collapse of hexagonal cells due to compression along the *x* direction, similar to the uniaxial buckling mode of the regular hexagonal honeycomb^20^,^26^. Interestingly, these buckling modes, which have been previously observed in the regular structure, did not lead to auxeticity.

**Figure 3:**
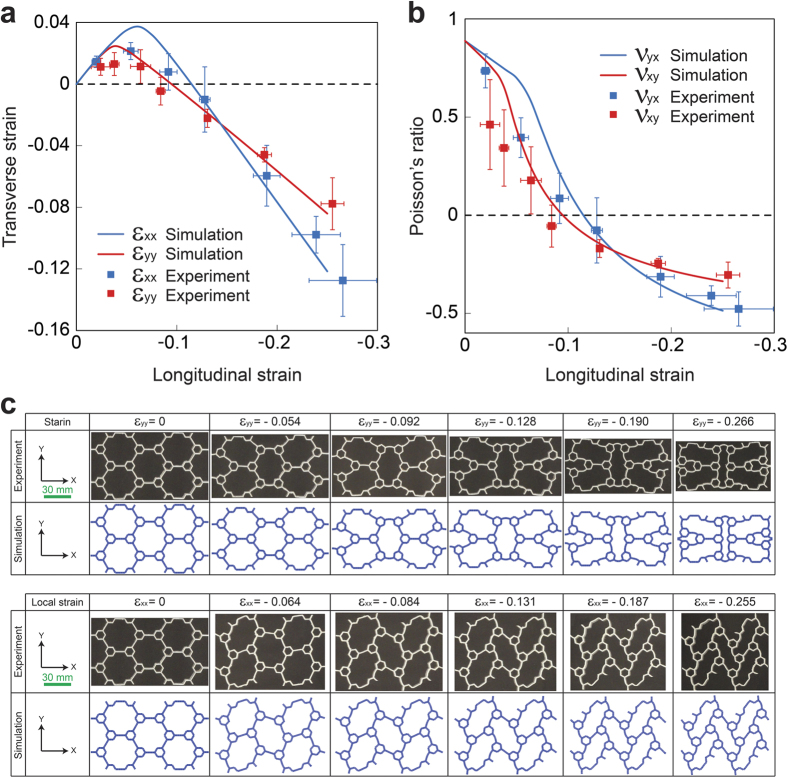
Validating the experiments using numerical simulations. (**a**) Transverse strain, and (**b**) Poisson’s ratio, versus longitudinal strain for both loading directions. The solid lines denote the simulation results and markers represent the experimental data. The error bars on the experimental points show the standard deviation of the values of the strain (and Poisson’s ratio) measured at different locations on the RVE. (**c**) Experimental and numerical images of deformed configuration of the RVE at different levels of deformation.

In order to quantify this behavior, we plot the transverse strain and Poisson’s ratio against the longitudinal strain for both loading directions, respectively in Fig. 3a,b, by post-processing photographs (See Supplementary Information for details on strain calculations). Next, we computationally analyzed a single RVE under uniaxial compression along the y and x direction using finite element (FE) simulations, intrinsically assuming the structure to be infinitely extended in 2D space (i.e., periodic boundary conditions were imposed)^18^. We first investigated the instability of the structure through a linear perturbation analysis^27^. Then, the non-linear post-buckling response of the system was simulated by introducing a small imperfection in the initial geometry (see Methods for more details). In Fig. 3a,b, FE results of the transverse strain and Poisson’s ratio (denoted by solid lines) are reported as functions of the longitudinal strain for both loading directions, which are in an excellent agreement with experimental results. Also, Fig. 3c compares the experimental and numerical images of deformed configurations of the RVE at different levels of applied compressive strain along the *y* (i.e., X-shape mode) and *x* (i.e., N-shape mode) directions, which are again in perfect agreement.

Although these plots confirm the difference in deformation behavior of the structure in the two directions, several important similarities exist. For instance, in both these directions, upon increasing the compressive strain, the transverse strain rises from zero (i.e., undeformed configuration) up to a turning point, and then decreases until it becomes zero at ~10% compressive strain, Fig. 3a. After this point, the transverse strain becomes negative showing lateral contraction (i.e., negative Poisson’s ratio). This similarity is further confirmed through Poisson’s ratio variation with longitudinal strain in two directions (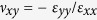 and 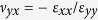), Fig. 3b. Note that small deformation Poisson’s ratios (~0.9) are in agreement with published literature^21^. The Poisson’s ratio decreases as the strain is increased: first, slowly as also seen for honeycombs with no hierarchy, and then follows by a sharp decrease due to instability. Negative Poisson’s ratio is achieved at ~10% compressive strain. Thereafter, the rate of reduction becomes smaller indicating the formation of a plateau regime with 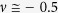 as the plateau Poisson’s ratio.

**Figure 4:**
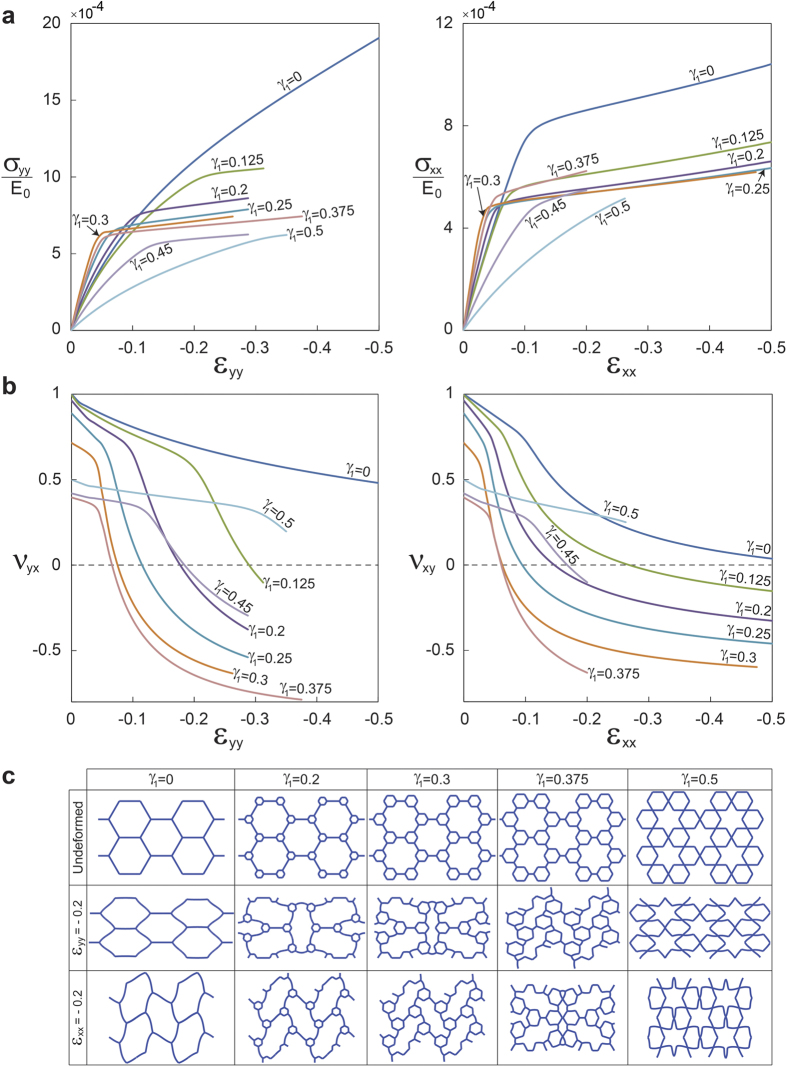
Strain-dependent response of first order hierarchical honeycombs. (**a**) Stress-strain curves, and (**b**) the evolution of Poisson’s ratio versus longitudinal strain for uniaxial compression along the *y* and *x* directions. The stress is normalized with respect to the initial Young’s modulus of the cell wall material (*E*_0_). (**c**) Undeformed and deformed configurations of the RVEs at 20% compression.

To further investigate the auxetic behavior of hierarchical honeycombs, we extend our validated simulations to study the effect of the parameter, *γ*_1_, on uniaxial compressive response of the structures with first order of hierarchy. Although no dynamic calculations were performed in this study, in order to isolate the effect of hierarchy, the relative density was kept constant at 8% for these simulations performed on the RVE as earlier. The results are presented for eight different values of *γ*_1_, varying from 0 (i.e., regular hexagonal honeycomb) to 0.5. Figure 4a plots the evolution of the normalized nominal stress, 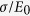 (where *E*_0_ is the initial Young’s modulus of the cell wall material), versus the applied longitudinal strain, *ε*. We find that no instability occurs in the structures with 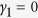 and 0.5 for compression along the *y* direction. In fact, the structures’ deformation is symmetrical and is formed by static deflection of the cell walls due to bending (see Fig. 4a (left) and 4c (middle row)). In contrast, for 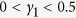, the response of the structure is characterized by a linear elastic regime followed by elastic buckling resulting in a stress plateau (i.e., a typical response for cellular solids). Similar phenomenon is observed for compression along the *x* direction (see Fig. 4a (right)) except for the structure with 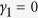 in which the elastic buckling (in contrast to the other loading direction) is observed (i.e., uniaxial mode of buckling of a regular hexagonal honeycomb (see Fig. 4c (bottom row))^26^).

**Figure 5:**
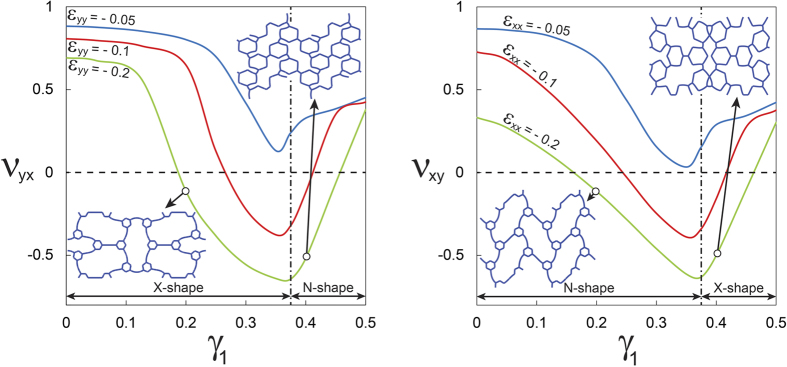
The evolution of Poisson’s ratio as a function of *γ*_1_ at 5, 10, and 20% compression along the *y* and *x* directions. The vertical dashed lines represent the point in which the Poisson’s ratio is minimum and the deformation mode switches.

Next, Fig. 4b which shows the evolution of Poisson’s ratio with longitudinal strain in both directions, indicates that in either direction of loading, for structures with no instability, Poisson’s ratio remains positive and smoothly decreases with deformation. On the other hand, Poisson’s ratio for the structures with elastic instability exhibits an initial slow decrease from the small deformation positive value transitioning to a negative regime via a sharp drop. Our simulations showed that this sharp transition can be further advanced by increasing *γ*_1_ which also lowers Poisson’s ratio for a given deformation. Interestingly, we find that this trend is abruptly arrested at around 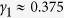, and then reversed for higher *γ*_1_ making it an important design parameter. Shedding greater light on this critical point, Fig. 4c, which shows the deformed configuration of the RVEs at 20% compressive strain for different geometries, reveals that the fundamental origin of this critical point is a switch in deformation mode at 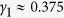 from X-shape to N-shape and vice versa for compression along the *y* and *x* directions, respectively. It is interesting to note that the *γ*_1_ corresponding to this critical point also results in the lowest possible Poisson’s ratio. Figure 5 displays the evolution of Poisson’s ratio against *γ*_1_ at 5, 10, and 20% longitudinal strain for both loading directions, showing that Poisson’s ratio reaches a minimum at 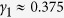, corresponding to the switching of the buckling modes.

**Figure 6:**
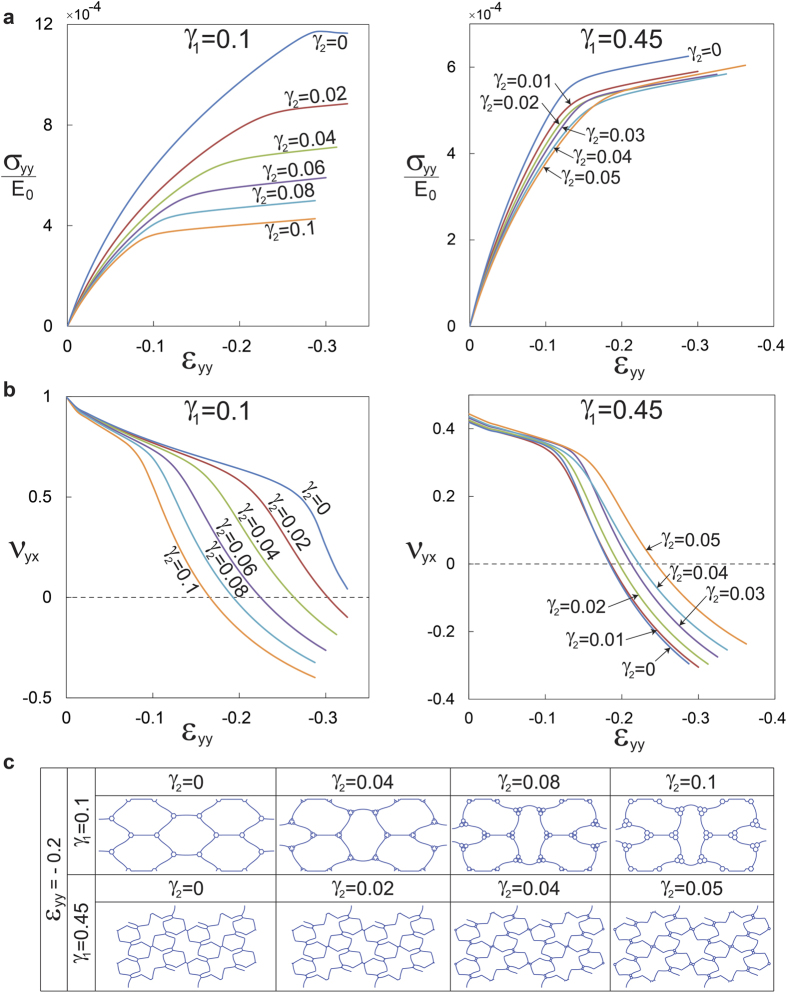
Honeycombs with second order of hierarchy. (**a**) Stress-strain curves, and (**b**) the evolution of Poisson’s ratio versus longitudinal strain, for second order hierarchical structures with 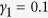 and 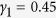, under uniaxial compression along the *y* direction (see Supplementary Information for loading along the *x* direction). The stress is normalized with respect to the initial Young’s modulus of the cell wall material (*E*_0_). (**c**) Deformed configuration of the RVEs at 20% compression.

Investigating the role of hierarchy further, we computationally study honeycombs with two orders of hierarchy with 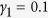 and 0.45 under uniaxial compression along the *y* direction (see Supplementary Information for loading along the *x* direction, exhibiting similar behavior). Carrying out FE simulations on RVEs with relative density held constant at 8%, we plot the normalized nominal stress against the applied strain, Fig. 6a. The effect of second order of hierarchy depends on *γ*_1_ values. For instance, the plateau stress decreases dramatically upon introducing second order of hierarchy into a structure with 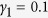 (see Fig. 6a (left)) in contrast to the structure with 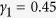 (see Fig. 6a (right)). More dramatically, second order of hierarchy can significantly advance auxeticity by significantly reducing the Poisson’s ratio with deformation for the 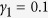 case (Fig. 6b (left)) whereas having an opposite effect for the 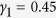 case. The contrasting behavior stems from the overall size of the smaller hexagons in the hierarchical structure, rather than from any fundamental change of buckling modes of the underlying structure due to introduction of the second order of hierarchy as confirmed in Fig. 6c. Essentially, introducing a higher order of hierarchy increases the overall size of the smaller hexagons, and this acts like increasing *γ*_1_ without increasing the order of hierarchy. Recalling our discussion from first order hierarchical structures, this makes the structures with *γ*_1_ less than the turning point value (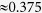) achieve a smaller Poisson’s ratio (moving left to right in Fig. 5) while the opposite being true for *γ*_1_ greater than this turning point value (also moving left to right in Fig. 5).

## Discussion and Conclusions

In summary, our experimental and computational study provides new insights on the behavior of auxetic metamaterials with structural hierarchy. We found that hierarchy-dependent elastic buckling introduced at relatively early stages of deformation decreases the value of Poisson’s ratio as the structure is compressed uniaxially leading to auxeticity in subsequent stages of deformation. This extraordinary behavior, which originates from structural hierarchy, has not been observed in the non-hierarchical regular structure, in spite of topical similarities in deformation modes. Our proposed hierarchical architecture is unique in exhibiting two different deformation modes for structures with different geometrical parameters when compressed along the same direction. An optimal design in terms of the lowest Poisson’s ratio is achieved among the structures with first order of hierarchy, which interestingly corresponds to a point in which the buckling modes switch. The auxetic response can further be pronounced (i.e., lower Poisson’s ratio) by introducing higher orders of hierarchy. Our results provide new insights into designing energy absorbing materials and tunable membrane filters^1^,^28^.

However, the main limitation of our proposed hierarchical architecture is the range of the strain at which the Poisson’s ratio becomes negative (i.e., critical strain ~10%). It is desirable to achieve auxetic behavior at much smaller strains for many practical applications. To reduce the critical strain, we tailor the geometry of the hierarchical structure for lowest critical strain. For instance, a hierarchical structure with first order of hierarchy has the lowest critical strain (~6%) for the geometry at which the switching of the buckling modes occurs and Poisson’s ratio attains the lowest value among all first order hierarchical structures. Interestingly, the critical strain can further be reduced by introducing higher orders of hierarchy as we demonstrated for second order of hierarchy.

## Methods

### Materials

A rubber-like flexible material (commercial name TangoGray, with material properties presented in Supplementary Information) was used to 3D print the experimental specimen. The material properties were measured through tensile testing on dog-bone specimens up to the strain of 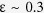. 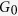 = 1.7 MPa and 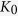 = 84.43 MPa are the initial shear and bulk moduli in the undeformed configuration and they are obtained by fitting the response under uniaxial tension of the bulk material (see Supplementary Information for more details).

### Fabrication using 3D printing

The specimen was fabricated using PolyJet 3D printing technique (Objet Eden260V 3D printer, Stratasys Inc., Eden Prairie, MN) out of TangoGray material. The specimen has overall size of Width × Hight × Depth = 254 × 229 × 20 mm with wall thickness of 1 mm, maintaining a relative density of 8% for 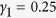. Prior to testing in either direction, two aluminum plates were attached to the top and bottom of the specimen to prevent the edge nodes from excessive bending.

### Mechanical testing

We applied uniaxial compression along the *y* and *x* directions using an Instron 5582 testing machine with a 1 KN load cell. In order to calculate the Poisson’s ratio at each level of applied compression, the photographs of deformed configurations of the specimen were recorded using a digital camera (See Supplementary Information for more details).

### Numerical simulations

The commercial FE package ABAQUS (SIMULIA, Providence, RI) was employed to carry out all the simulations in this study. Both microscopic and macroscopic instability analyses as well as post-buckling analysis were performed using the ABAQUS/Standard solver. The 2D FE models of periodic unit cells for the first and second order of hierarchical honeycomb were constructed using beam elements (ABAQUS hybrid element type B22H) and the accuracy of the mesh is insured by a mesh refinement study. The models were subjected to uniaxial static compression along the *y* and *x* directions while the lateral contractions were monitored. The first four eigenvalues from the instability analysis were employed to model imperfections in non-linear post-buckling analysis.

## Additional Information

**How to cite this article**: Mousanezhad, D. *et al.* Hierarchical honeycomb auxetic metamaterials. *Sci. Rep.* **5**, 18306; doi: 10.1038/srep18306 (2015).

## Supplementary Material

Supplementary Information

## Figures and Tables

**Figure 1:**
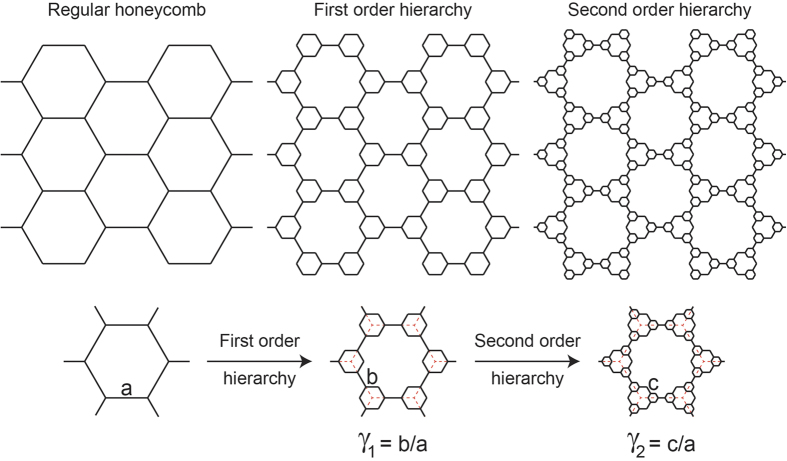
Schematic showing the evolution of a regular hexagonal honeycomb and its corresponding cell into first and second orders of hierarchy. The structural organization of the hierarchical structure at each order of hierarchy (*γ*_*i*_) is defined as the ratio of the newly added hexagonal edge length (b for first order and c for second order of hierarchy) to the original hexagon’s edge length (a) (i.e., 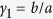 and 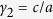)^21^.
