# Supplementary material for: Hierarchical honeycomb auxetic metamaterials
Source: Sci Rep. 2015 Dec 16;5:18306. doi: 10.1038/srep18306 (PMC4680941; doi:10.1038/srep18306)
Supplement: Supplementary Information [file srep18306-s1.pdf]

Supplementary Information

Hierarchical honeycomb auxetic metamaterials

Davood Mousanezhad[1], Sahab Babaee[2], Hamid Ebrahimi[1], Ranajay Ghosh[1],

Abdelmagid Salem Hamouda[3], Katia Bertoldi[2], and Ashkan Vaziri[1,*]

[1]Department of Mechanical and Industrial Engineering,

Northeastern University, Boston, MA 02115, USA

[2]Harvard John A. Paulson School of Engineering and Applied Sciences,

Harvard University, Cambridge, MA 02138, USA

[3]Mechanical and Industrial Engineering Department,

Qatar University, Doha, Qatar

Correspondence and request for materials should be addressed to A.V. (vaziri@coe.neu.edu)

Hierarchical honeycombs

The first order of hierarchy is obtained by replacing all three-edge nodes of a regular hexagonal honeycomb with smaller, parallel hexagons. This procedure can be repeated at smaller scales to achieve higher orders of hierarchy, yet the thickness of the cell walls must be reduced simultaneously to keep the overall density fixed. Figure 1 shows the evolution of a regular hexagonal honeycomb and its corresponding cell as the order of hierarchy is increased.

The structural organization of the structure at each order of hierarchy (γ_i) is defined by the ratio of the newly added hexagonal edge length (b for first order and c for second order of hierarchy)

(see Fig. 1) to the original hexagon's edge length (a) (i.e., $\gamma_1 = b/a$ and $\gamma_2 = c/a$)[1]. For first order of hierarchy, $0 \le b \le a/2$ and thus, $0 \le \gamma_1 \le 0.5$, where $\gamma_1 = 0$ represents the regular hexagonal honeycomb. For a structure with second order of hierarchy, $0 \le c \le b$ and $c \le a/2 - b$ and thus, $0 \le \gamma_2 \le \gamma_1$ if $\gamma_1 \le 0.25$, and $0 \le \gamma_2 \le (0.5 - \gamma_1)$ if $0.25 \le \gamma_1 \le 0.5$. These geometrical constraints must be imposed on the structures to avoid overlapping edges. The dimensionless relative density (i.e., area fraction), compared to the material density, is given as:

$$\rho = 2/\sqrt{3}\ .(1 + 2\gamma_1 + 6\gamma_2)\ .t/a \qquad \text{(S1)}$$

where t is the wall thickness which is assumed to be uniform throughout the structure.

Experimental specimen - material properties

We used a rubber-like flexible material (commercial name TangoGray) as the bulk material to fabricate the specimen. To measure the material properties, five dog-bone specimens were 3D printed and tested under uniaxial tensile loading, and the stress-strain response of the material (i.e., engineering stress vs. engineering strain) is monitored up to the strain of $\varepsilon = 0.3$ (see Fig. S1). Since the test was performed quasi-statically, we neglected viscoelastic effect and captured the constitutive behavior using a nearly-incompressible Neo-Hookean hyperelastic model (Poisson's ratio: $\nu_0 = 0.49$), whose strain energy is given by $U = G_0(\bar{I}_1 - 3)/2 + K_0(J-1)^2/2$, where $\bar{I}_1 = J^{-2/3}\ \mathrm{tr}[\mathrm{dev}(\mathbf{F}^{\mathrm{T}}\mathbf{F})]$, $\mathrm{J} = \det(\mathbf{F})$, and $\mathbf{F}$ is the deformation gradient. We obtained the initial shear modulus (G_0=1.7 MPa) and bulk modulus (K_0=84.43 MPa) in the undeformed configuration by fitting the material model to the experimental data (see Fig. S1). The mechanical tests were performed based on ASTM-D638-10, which is the standard test method for measuring tensile properties of plastics.

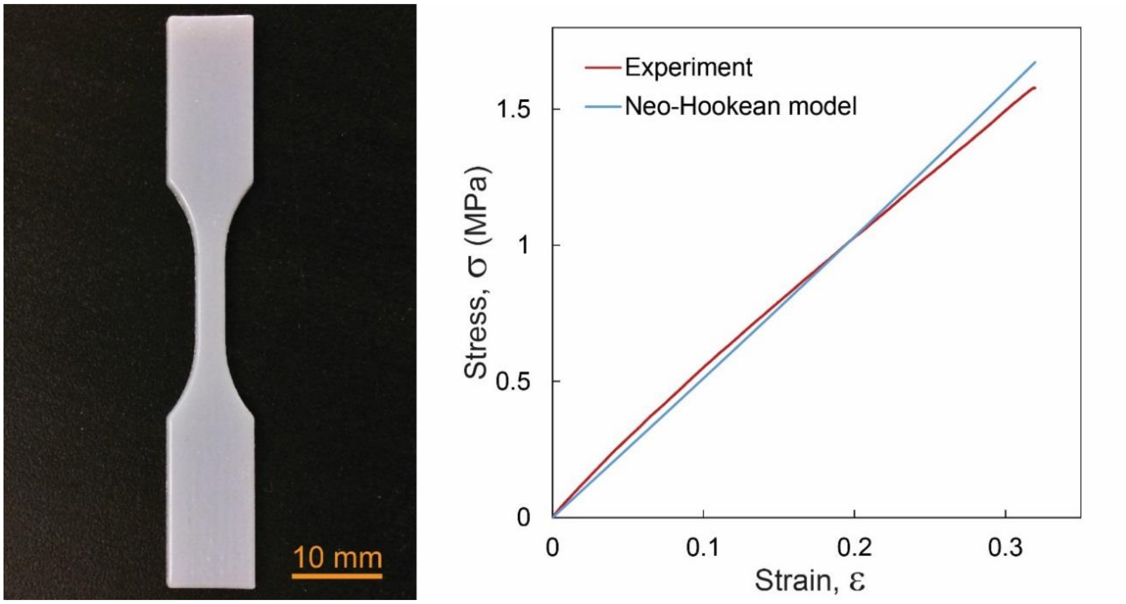

Figure S1. (Left) 3D printed dog-bone specimen for uniaxial tension test. (Right) Engineering stress vs. engineering strain for the tested material.

Experiments

The specimen was fabricated using PolyJet 3D printing technique out of TangoGray material which has an overall size of Width × Hight × Depth = 254 × 229 × 20 mm with wall thickness of 1 mm, maintaining a relative density of 8% for $\gamma_1 = 0.25$. Next, we applied uniaxial compression along the *y* and *x* directions using an Instron 5582 testing machine with a 1 KN load cell. In order to calculate the Poisson's ratio at each level of applied compression, the photographs of deformed configurations of the specimen was recorded using a digital camera. Then, we tracked RVEs' boundary points (i.e., the points in touch with adjacent RVEs) to obtain their locations at different levels of deformation and used them to measure the horizontal and vertical distances (i.e., Δx and Δy, see Fig. S2) needed to evaluate the local strains (i.e., $\varepsilon_{xx} = \Delta x/\Delta x(0)$ and $\varepsilon_{yy} = \Delta y/\Delta y(0)$). The measurement was repeated at least at five different locations on the RVE at each

level of compression. The Poisson's ratio is then calculated as $\nu_{xy} = -\varepsilon_{yy}/\varepsilon_{xx}$ and $\nu_{yx} = -\varepsilon_{xx}/\varepsilon_{yy}$.

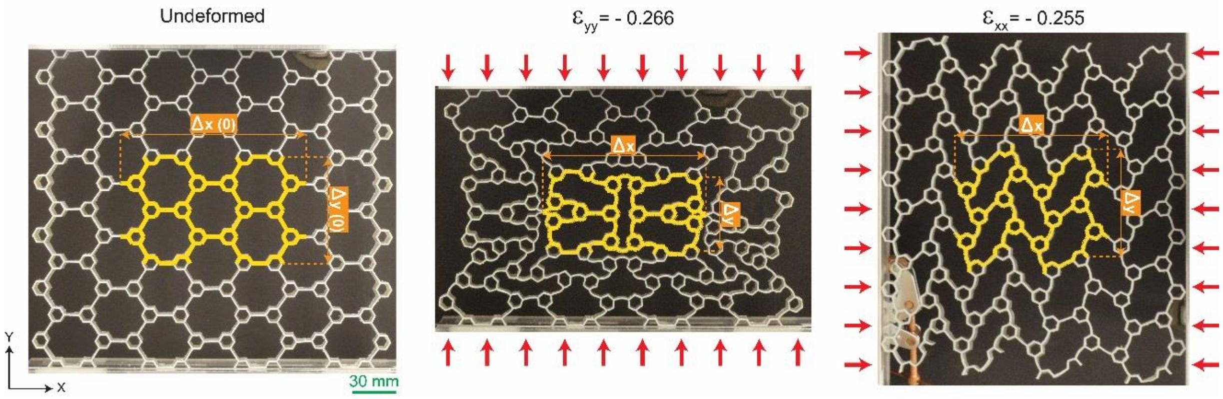

Figure S2. Hierarchical honeycomb auxetic metamaterials: experimental setup. (left) Undeformed configuration of the fabricated first order hierarchical structure with $\gamma_1 = 0.25$. The representative volume element (RVE) is highlighted as yellow. (middle and right) Deformed configurations of the specimen and the RVE under compression along the *y* ($\varepsilon_{yy} = -0.266$, X-shape deformation mode) and *x* ($\varepsilon_{xx} = -0.255$, N-shape deformation mode) directions, respectively.

Second order of hierarchy – additional results

We performed FE simulations on second order hierarchical structures with $\gamma_1 = 0.1$ and 0.45 under uniaxial compression along the *x* direction. The relative density was kept constant at 8%. The results are presented in Fig. S3 in the form of the normalized nominal stress (Fig. S3a) and Poisson's ratio (Fig. S3b) versus the applied compressive strain. Deformed configuration of the RVEs at 20% compressive strain is also presented in Fig. S3c. The results presented in Fig. S3, which show the effect of the second order of hierarchy on auxetic response of hierarchical honeycombs, are similar to the results presented in Fig. 6 for compression in y direction.

Next, Fig. S4 plots the evolution of Poisson's ratio against γ_2 at 20% compressive strain for second order hierarchical structures with $\gamma_1 = 0.1$ and 0.45. The figure clearly shows that second order of hierarchy reduces the value of Poisson's ratio for the structure with $\gamma_1 = 0.1$, whereas the opposite is true for the structure with $\gamma_1 = 0.45$. The reason for this behavior relies on the overall size of the smaller hexagons in the hierarchical structure. Introducing a higher order of hierarchy increases the overall size of the smaller hexagons, and this acts like increasing the value of γ_1 without increasing the order of hierarchy. This makes the structures with $\gamma_1 < 0.375$ to achieve a smaller Poisson's ratio (moving from left to right in Fig. 5) with the opposite being true for $\gamma_1 > 0.375$ (moving from left to right in Fig. 5).

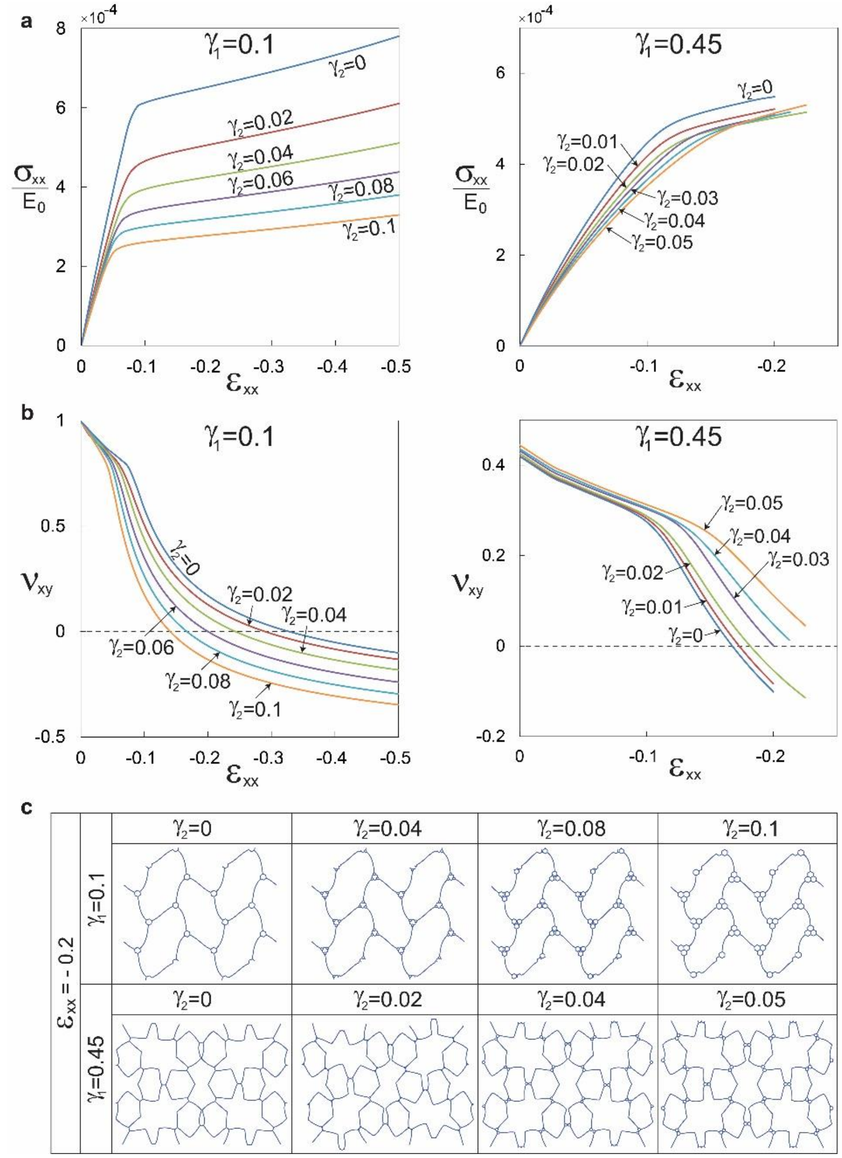

Figure S3. Honeycombs with second order of hierarchy. a) Stress-strain curves, and b) the evolution of Poisson's ratio versus longitudinal strain, for second order hierarchical structure with $\gamma_1 = 0.1$ and $\gamma_1 = 0.45$, under uniaxial compression along the x direction. The stress is normalized with respect to the initial Young's modulus of the cell wall material (E_0). c) Deformed configuration of the RVEs at 20%.

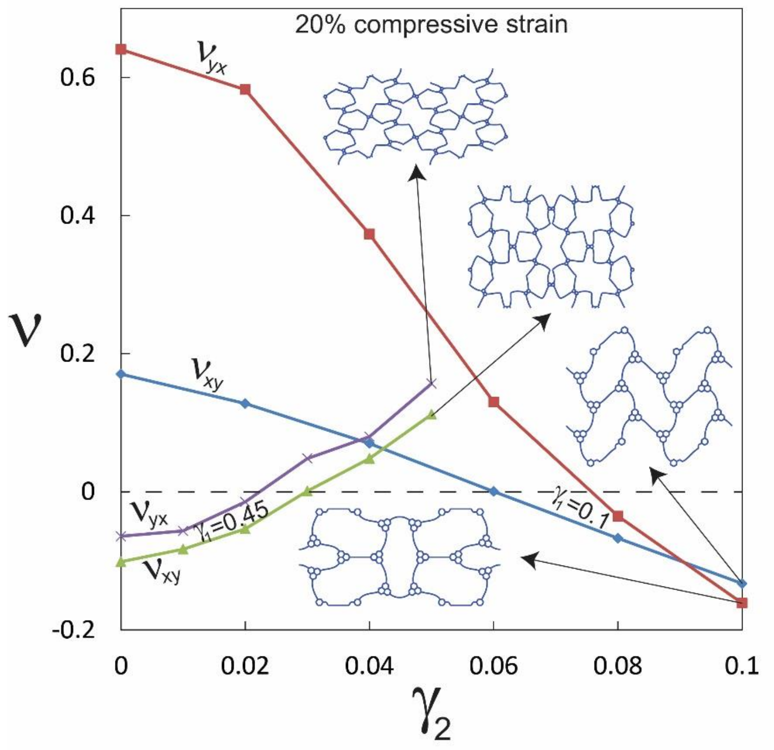

Figure S4. Poisson's ratio vs. γ_2 for second order hierarchical structures with γ_1=0.1 and 0.45.

References

1 Ajdari, A., Jahromi, B. H., Papadopoulos, J., Nayeb-Hashemi, H. & Vaziri, A. Hierarchical honeycombs with tailorable properties. *International Journal of Solids and Structures* **49**, 1413-1419, doi:http://dx.doi.org/10.1016/j.ijsolstr.2012.02.029 (2012).
